# Taxonomical Identification and Safety Characterization of *Lactobacillaceae* from Mediterranean Natural Fermented Sausages

**DOI:** 10.3390/foods11182776

**Published:** 2022-09-09

**Authors:** Daniela Bassi, Giovanni Milani, Mireya Viviana Belloso Daza, Federica Barbieri, Chiara Montanari, Silvia Lorenzini, Vida Šimat, Fausto Gardini, Giulia Tabanelli

**Affiliations:** 1 Department for Sustainable Food Process (DISTAS), Università Cattolica del Sacro Cuore, 26100 Cremona, Italy; 2 Department of Agricultural and Food Sciences, University of Bologna, 40127 Bologna, Italy; 3 University Department of Marine Studies, University of Split, Ruđera Boškovića 37, HR-21000 Split, Croatia; 4 Interdepartmental Center for Industrial Agri-Food Research, University of Bologna, 47521 Cesena, Italy

**Keywords:** *Lactobacillaceae*, spontaneously fermented sausages, Mediterranean Countries, safety assessment

## Abstract

Fermented meat products represent an important industrial sector in Europe, particularly in the Mediterranean Countries (MC), where the presence of numerous local productions, still obtained through spontaneous fermentation, is recognized as a formidable treasure chest of unexplored microbial biodiversity. *Lactobacillaceae* naturally occurring in fifteen spontaneously fermented sausages from MC (Italy, Spain, Croatia, and Slovenia) were isolated and taxonomically characterized using molecular techniques. Additionally, a safety assessment for the presence of antibiotic resistances and biogenic amine (BA) production was performed to determine their suitability as autochthonous starter cultures. Molecular typing, performed using REP-PCR, discriminated 151 strains belonging to *Latilactobacillus sakei* (59.6%), *Latilactobacillus curvatus* (26.5%) and *Companilactobacillus alimentarius* (13.9%). The minimum inhibitory concentrations (MICs) of eight different antibiotics revealed a high resistance to streptomycin (27%), tetracycline (16%), followed by gentamycin (14%) and kanamycin (13%). Interestingly, the results showed a geographical distribution of resistant biotypes. *tetM*/*tetS* or *ermB* genes were identified in only six strains. The amino-biogenic potential of the strains was assessed, confirming the absence of this trait among *L. sakei*, while a high number of producer strains was found among *L. curvatus*. On the 151 analyzed strains, 45 demonstrated safety traits for their future use as starter food cultures. These results open the way to further studies on the technological properties of these promising autochthonous strains, strongly linked to the Mediterranean environment.

## 1. Introduction

The curing of meats can be considered one of the most ancient methods for preserving perishable raw materials and the origin of this approach dates back several centuries [1,2]. Among cured meats, dry sausage preparation combines the use of curing salts with a fermentation step that involves several microorganisms including lactic acid bacteria (LAB), staphylococci, micrococci and fungi [3,4]. Early studies concerning the complex dynamics of this microbial process were published about 60 years ago [5,6] and lead to establish criteria f or the selection of starter cultures to be used for driving meat fermentation [7,8]. Nowadays, the use of starter cultures is common in industrial products, and they are mainly constituted of LAB and coagulase negative cocci (CNC). Among LAB, the strains mainly selected belong to the species *Latilactobacillus sakei*, *Latilactobacillus curvatus*, *Lactiplantibacillus plantarum*, *Pediococcus pentosaceus* and *Pediococcus acidilactici* [9]. However, as observed by [2], the use of starters may result in the loss of microbial biodiversity and, therefore, of the peculiar characteristics (impoverishment of sensory features), if compared to artisanal sausages obtained with spontaneous fermentation. On the other hand, selected cultures are able to guarantee the constant quality, safety and longer shelf-life [2,10]. The search for new and tailor-made cultures able to impart specific and traditional attributes to fermented sausages represents an important approach to overcome the negative aspects and preserving the authenticity and recognisability of artisanal products. In this perspective, the presence of numerous local products still obtained through spontaneous fermentation is a formidable source of unexplored microbial biodiversity of a given terroir which could be exploited for isolating new starter candidates [11,12,13].

The most peculiar traits of LAB strains in meat fermentation are undoubtedly their abilities to rapidly decrease the pH and to colonize the environment throughout the complete production process [14]. A low pH limits the growth of undesirable species (pathogens and spoilers) and favours texture and water loss by approaching the isoelectric point of meat proteins [15]. However, further technological characteristics are requested for their use as meat starter cultures. These features can represent an important trait for starter selection also in the frame of NaCl reduction, which characterizes the new market trends considering nutritional needs and consumer demands [16]. Generally, LAB starters are selected in order to improve safety and reduce hygienic and toxicological risks in food [2,17], but they have to be firstly safe, so that their use as starter cultures for fermentation requires a safety assessment. Biogenic amines (BA) are toxic products deriving from amino acid decarboxylation that accumulate in sausages during fermentation and ripening [18]. Many bacterial species may contribute to their production in fermented foods, including LAB. For this reason, the absence of specific decarboxylases is a prerequisite for LAB used as starter cultures, mainly because their performances could affect BA accumulation during ripening [19,20]. The presence of genetic clusters containing the necessary genes for BA production have been deeply studied, especially for the most dangerous BA, i.e., tyramine and histamine [21,22]. The conditions of BA accumulation define the so called aminobiogenic potential, that can be tested both genetically and phenotypically [23]. Another relevant safety aspect is the presence of antibiotic resistance genes in mobile genetic elements, such as plasmids and transposons. In fact, these elements can be transferred to other species, including pathogenic bacteria, during food manufacture or during the passage through the gastrointestinal tract [24]. This poses an additional risk due to the nature of consumption of ready-to-eat fermented products and their potential to become strong antibiotic resistance reservoirs [25]. Particularly, the presence of tetracycline and erythromycin resistant lactobacilli, studied applying EFSA (European Food Safety Authority (EFSA) 2012) cut-off limits, has been well documented in fermented dry sausages produced in northern Italy [26,27].

The aim of this study was the isolation, characterization and safety assessment of autochthonous *Lactobacillaceae* from 15 Mediterranean spontaneously fermented sausages, collected from four different MC (Italy, Spain, Croatia, and Slovenia) and previously characterized for their characteristics and bacterial biodiversity [28]. This investigation had the purpose to widen the previously studied microbiota composition, in order to understand the ecology of these natural fermented meats and to know which LAB species are the most abundant. In addition, the work was aimed to study the presence and the type of strain antimicrobial resistances and aminobiogenic potential. With this aim, more than 900 isolates have been genotyped using fingerprint analysis for the differentiation of the strains, which have been further taxonomically identified and characterized for their safety features. This knowledge will be the starting point to further determine strains suitability to be used in foods from one side as potential autochthonous starter cultures, studying their technological properties, and secondary, as protective food cultures against pathogenic and spoiling agents, assessing their antimicrobial potential.

## 2. Materials and Methods

### 2.1. Sausage Samples and Lactobacillaceae Isolation

A sampling of 15 natural-fermented sausages, produced without any starters addition, was collected at the end of ripening from four different MC and particularly: three sausages were obtained from Italy (IM1, IM2, IAL), two from Slovenia (SN, SWO), seven from Spain (ESA, ESB, ESE, ESO, ECB, ECE, ECO) and three from Croatia (HNS, HS, HZK).

Times and ripening conditions were heterogeneous and only for the three Slovenian domestic samples, smoking was used (14 days). These samples were previously characterized for their chemical-physical features and their microbial profile [28].

For cultivation-dependent analysis, sausages were processed as previously described by Barbieri et al. [28]: Man-Rogosa-Sharpe (MRS) agar medium (Oxoid, Milan, Italy) was employed for presumptive LAB counts and their isolation at 30 °C for 48 h in anaerobic conditions achieved using Anaerocult A (Merck, Darmstadt, Germany) in anaerobic jars. Grown colonies on MRS agar plates were randomly selected, picked with a sterile loop, and streaked onto new MRS plates in duplicate. Isolates were collected from plates containing from 20 to 50 colonies. A minimum of 22 to a maximum of 70 isolates for each sample was considered. The pure cultures obtained were observed for morphological characteristics and tested by means of catalase test, Gram test, growth at 15 °C and 45 °C, as well as for their homo or heterolactic fermentation. For further analyses, the isolates were stored at −20 °C in MRS broth containing 20% glycerol (Carlo Erba, Milan, Italy).

### 2.2. DNA Extraction and REP-PCR Analysis

All isolates were cultured in 10 mL of MRS broth (GranuCult^®^, Darmstadt, Germany), incubated at 30 °C overnight. From these fresh pure cultures, isolates were streaked in MRS agar and incubated in anaerobic conditions for 48 h. Single colonies were selected from the agar plates to perform DNA extraction using the fast microLYSIS^®^-Plus DNA extraction kit (Microzone, Labogen, Stourbridge, UK) following the manufacturer’s instructions; 20 µL of DNA was obtained and used for the molecular analysis. The rep-PCR (Repetitive element (or extragenic) palindromic-Polymerase Chain Reaction) using oligonucleotide primer (GTG) 5 (5′–GTGGTGGTGGTGGTG–3′), was chosen for fingerprint analysis of isolates [29]. The amplified products were electrophoresed in a 2.5% agarose gel. The study was performed analysing the fingerprint profile for each group of isolates from the different sausage types. The selected biotypes were grown in 10 mL of MRS broth and incubated overnight at 30 °C under anaerobic conditions. Cells were collected by centrifugation (3200× *g*, 15 min) and frozen at −20 °C in MRS + glycerol 20% solution.

### 2.3. Genotyping Identification of Isolates

16S rRNA gene sequencing was done on all biotypes with different rep-PCR profiles using specific primers and PCR reaction designed by [30]. After amplification and before sequencing, the PCR products were purified using ExoSAP-IT™ (Applied Biosystems™, Thermo Fisher Scientific, Leicestershire, UK) according to the protocol provided by the manufacturer. The DNA was sequenced by a commercial facility (Eurofins Genomics, Italy) and the obtained sequences were analysed using the Ribosomal Database Project tools (http://rdp.cme.msu.edu/, accessed on 1 Febraury 2021) and assigned to the species with the highest percentage of identity. Species-specific PCR reactions for the identification of *Latilactobacillus sakei* and *Latilactobacillus curvatus* were performed on those isolates that were not correctly assigned to species level (identity ≤ 98.7% [31]). PCR products were separated by electrophoresis in a 1% agarose gel and visualized by Sybr-Safe staining.

The relative frequency of intra-species biotypes for each MC salami sample has been calculated using Microsoft Excel 2016, Version 2207.

### 2.4. Antibiotic Susceptibility Testing

EFSA Guidance [32] was followed for antibiotic resistance determination of the selected strains and minimal inhibitory concentration (MIC) testing. Resistances to Ampicillin (Amp), Chloramphenicol (Chl), Clindamycin (Cli), Erythromycin (Ery), Gentamicin (Gen), Tetracycline (Tet), Kanamycin (Kan) and Streptomycin (Str) were determined by using micro dilution technique in the recommended LSM medium (Iso-Sensitest™ broth 90% [Thermo Fisher Scientific, Leicestershire, UK], MRS broth 10%) [33]. Bacterial growth was measured after 48 h of incubation at 28 °C for *Latilactobacillus sakei* and 37 °C for *Latilactobacillus curvatus*, under anaerobic conditions.

Relative abundance of resistant biotypes for each species and antibiotics tested were calculated using Microsoft Excel 2016, Version 2207 (Microsoft Corporation, Redmond, WA, USA).

### 2.5. PCR-Based Screening of Resistance Genes

The presence of Tetracycline and Erythromycin resistance genes was screened by standard PCR with specific primers reported in Table 1. Genes coding for ribosomal protection proteins conferring Tetracycline resistance were targeted with specific primers for *tet* (*W*) [34], *tet* (*M*) [35] and tet (S) [36]. Tetracycline efflux pump gene, *tet* (*L*) was also searched using gene-specific primers [37]. The presence of Erythromycin resistance genes was tested using specific primers for *ermA*, *ermB* [38] and *ermC* [39]. PCR products were separated by electrophoresis on a 1% agarose gel and visualized by Sybr-Safe staining.

### 2.6. Biogenic Amines (BAs) Production

To test the aminobiogenic potential of the strains, overnight cultures on MRS were inoculated to a concentration of 6 log CFU/mL in Bover-Cid and Holzapfel broth, supplemented with the BA precursors (histidine, tyrosine, ornithine or lysine) and incubated at 30 °C. The ability to produce BAs was assessed with the method proposed by Bover-Cid and Holzapfel (1999) and BA production confirmation was performed thought HPLC according to the method reported by [28].

## 3. Results and Discussion

### 3.1. Strain Genotyping and Identification of Biotypes

The spontaneously fermented sausages used as a source for the selection of new LAB strains were produced according to traditional local recipes and varied with regards to the ingredients, the presence of additives, the casing, the fat, and the ripening conditions as described by [28].

A total of 914 microorganisms, grown on MRS agar medium and presumptive classified as LAB based on Gram staining and catalase test results, were isolated from 15 natural-fermented sausages from MC: 173 isolates from Italian sausages, 140 from Slovenian sausages, 444 from Spanish sausages and 157 from Croatian sausages, respectively. To achieve taxonomical identification at the strain level, (GTG)5-rep-PCR fingerprinting technique was applied on DNA extracted from the all the isolated samples. Representative profiles for each sausage were selected and subjected to partial 16S rRNA gene sequencing and species-specific PCR for *L. sakei* and *L. curvatus*. In Table 2 the total number of isolates and biotypes per each type of MC sausage are reported.

Based on the data described in Table 2, a total of 151 biotypes were detected. The fingerprint analysis showed a higher variability in the electrophoretic profiles of the samples from Spanish and Croatian sausages. From 69 and 70 isolates of the Spanish Salchichones ESB and ESO, 11 and 19 different biotypes have been respectively differentiated, while out of 48 and 69 isolates from the Spanish Chorizo ECB and ECE, 16 and 22 biotypes have been highlighted. Regarding Croatian samples, the richest in LAB biodiversity was the Salami HZK, with 21 biotypes out of 53 isolates. Differently, Italian and Slovenian sausages were characterized by a lower biodiversity, with only two identified biotypes among 48 isolates from Italian Salame IM2, 5 biotypes among 70 isolates from the Slovenian traditional smoked salami SN and 6 biotypes among 70 isolates from the Slovenian traditional smoked salami SWO.

Secondarily, the 151 strains that showed unique rep-PCR profiles, were identified by 16S rRNA gene sequencing. Isolates that failed to be assigned to any species (level of identity ≤ 98.7%), were identified using species-specific PCR for the identification of *L. sakei* and *L. curvatus*. The combined molecular approach allowed to identify three dominant species: *L. sakei*, *L. curvatus* and *C. alimentarius*; particularly, 90 strains were assigned to *L. sakei*, 40 strains to *L. curvatus* and 21 strains to *C. alimentarius*. As frequently stated by previous works [40,41], natural meat fermentation is dominated by coagulase-negative cocci (CNC) and LAB, whose most commonly species are represented by *L. sakei*, *L. curvatus* and *L. plantarum*. Considering the results of the combined methodology to assess the taxonomic identity of the 150 *Lactobacillaceae* biotypes, *L. sakei* resulted to be the dominant species in the IM1 (100%), IM2 (100%), IAL (75%), SN (100%), SWO (100%), ESB (91%), ESE (62.5%), ESA (82%), HZK (81%), HS (100%), HNS (64%) sausages. *L. curvatus* prevailed in the ECE (63.7%) sausages, while the ESO sausages presented an equal presence of *L. sakei* and *L. curvatus* (47% each). *C. alimentarius* dominated in the ECB (87.5%) and in the ECO (57%) sausages.

**Figure 1:**
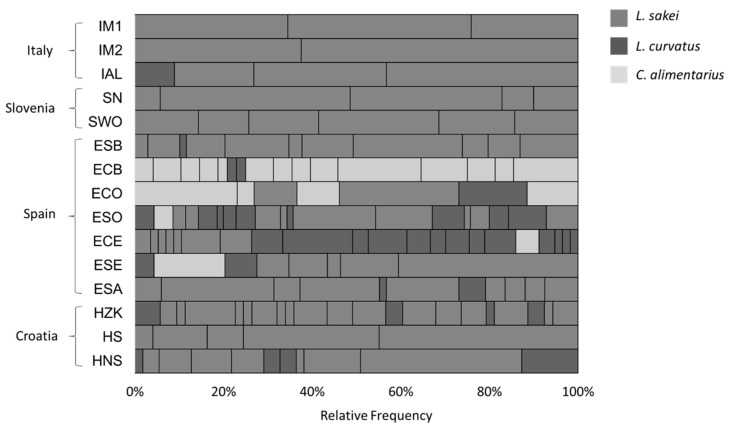
Relative frequency of LAB biotypes, identified as *L. sakei*, *L. curvatus* and *C. alimentarius*, found in each MC fermented sausages.

These data confirmed the outcome obtained through metagenomics analysis on the same samples [28], where *L. sakei* was the dominant species among LAB, especially in IM2, IAL, and SN samples, followed by members of the *L. sakei* group in lower amounts. This dominance is highlighted also by the relative frequency of the *L. sakei* biotypes that were found in higher percentage particularly in the Italian and Slovenian sausages, followed by Croatian ones; a higher species biodiversity was instead typical of Spanish samples, where the species distribution among biotypes is more balanced for the three described LAB (Figure 1 and Supplementary Material Table S1).

During the ripening process of natural fermented sausages, LAB species diversity is limited and *L. sakei* is one of the main adapted species to the restrictive conditions generally present in the dry meat environment, due to the species excellent adaptation, competitiveness and assertiveness in the meat matrix [12,42]. The samples of the study were processed at the end of the maturation period when low availability of sugars was still supposed to be present in the sausages and free amino acids were probably used by *L. sakei* to grow and survive in these conditions. This species is highly adapted to this ecological niche, due to its metabolic pathways, including the arginine deiminase pathway and the utilization of nucleosides [12,43,44].

The Spanish sausages surprisingly showed a high percentage of biotypes belonging to the *C. alimentarius* species; sample ECB showed, for example, 95% *C. alimentarius* biotypes. *C. alimentarius* species was also previously highlighted through amplicon sequencing and metagenomic analysis in these Mediterranean sausage samples [28]. In fact, also in this preliminary work, high quantities of the members of *C. alimentarius*, *C. heilongjiangensis* and *C. versmoldensis* were present in many Spanish sausages, in particular ESE and ECB (55.3 and 45.0% of the total ASVs, respectively). This species has been reported as a regional peculiarity in the literature [45,46], but its presence was described also as minoritarian in some traditional fermented salami of Southern Italy, such as Naples-type salami [47]. In addition, the Spanish sausages, if compared to the other MC fermented meats, had a higher strain biodiversity in terms of different identified biotypes. The technological parameters, together with the ingredients, the fermentation process and ripening conditions could strongly influence the survival and adaptation of different bacterial populations to a peculiar environment.

### 3.2. Safety Assessment of Isolated Strains

Starter strains to be used as food cultures have to comply with safety criteria such as the absence of antibiotic resistance genes and the incapacity to produce biogenic amines [48,49]. In this context, the two main occurring species *L. sakei* and *L. curvatus* (90 and 39 isolates, respectively) were tested for their safety features in order to prove their safe use as food cultures. Particularly, their antimicrobial resistance profile was tested by micro dilution technique and the aminobiogenic potential through HPLC analysis. *C. alimentarius* strains were not analysed for the characterization, since this species is not considered among the possible adequate starter cultures in meat productions.

#### Antibiotic Resistance Assessment

Cut off values established by EFSA [32], were used as reference to search for the presence of resistant biotypes isolated from the 15 artisanal fermented sausages. A unimodal distribution of the MICs values, divided per species, is reported in Table 3 for all the analysed samples.

Considering *L. sakei*, the most representative isolated species, 45 strains out of 90, showed resistance to at least one antibiotic. A total of 28 strains presented only 1 resistance, while a discrete number of multidrug resistant strains have been found; particularly, 11 strains showed 2 resistances, 5 strains carried 3 resistances and 1 strain, the ECE-5 isolated from a Spanish sausage, presented 5 resistances (data not shown). For what concerns the antibiotic classes, these results demonstrated that there is a limited susceptibility to aminoglycosides, especially for Streptomycin, with 25 resistant strains, followed by Tetracycline, with 15 resistant strains, Gentamycin, with 13 resistant strains, and by Kanamycin, with 11 resistant strains. The genotypic analysis on *L. sakei* showed the presence of two genes coding for Tetracycline resistance: *tetS* and *tetM*. *TetS* gene was detected in the strains HNS-7, HNS-3 and ESB-57, while *tetM* gene was found in HNS-3, ECO-19 and ESB-57. No resistant strains were detected for Erythromycin and Ampicillin. In the case of *L. curvatus*, 20 out of the 40 strains were recognised as resistant. Especially, 12 strains showed one resistance, only one strain presented 2 resistances, 6 strains carried 3 resistances and 1 strain, the HZK-49 isolated from a Croatian sausage showed 5 resistances. In this case, the results showed that susceptibility to aminoglycosides is the lowest; particularly, 11 strains presented resistance to Streptomycin, followed by Gentamycin, with 5 resistant strains, and by the Kanamycin, with 3 resistant strains. In addition, 7 isolates were resistant to Tetracycline, 4 isolates were resistant to Chloramphenicol, 3 strains were resistant to Clindamycin and 2 strains were resistant to Ampicillin. Furthermore, the genotypic analysis presented the gene *ermB* coding for Erythromycin resistance in *L. curvatus* strains ESO-52 and HZK-49; the results confirmed the output obtained with the micro dilution method.

**Figure 2:**
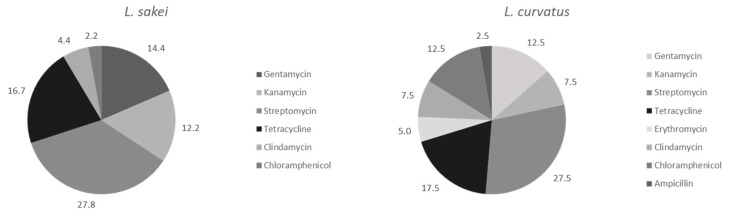
Relative abundance of resistant biotypes for each species and antibiotics tested.

Comparing the two LAB dominant species for antibiotic resistance, Streptomycin resulted to be the most spread resistance both in *L. sakei* and *L. curvatus* isolates (Figure 2), followed by Tetracycline. Differently from *L. curvatus* strains, *L. sakei* isolates harboured no resistances to Ampicillin and Erythromycin.

The occurrence of strains characterized by MICs higher than EFSA breakpoints was found to be superior in the ones isolated from the Spanish and the Croatian sausage samples, showing a geographical distribution of resistant biotypes. In fact, the highest values for Gentamycin was 64 µg/mL (ESA and ESE sausages); for Kanamycin was 256 µg/mL (ESA, HZK, HS sausages); for Streptomycin was 256 µg/mL (ESO, ECE, ESE, HZK, HNS sausages); for Tetracycline was 64 µg/mL (ESB, ECO, ESO, ECE, HZK and HNS sausages); for Erythromycin was 2 µg/mL (ESO, HZK sausages); for Clindamycin was 8 µg/mL (HS sausage); for Chloramphenicol was 8 µg/mL (ECE and HZK sausages); for Ampicillin was 8 µg/mL (ESO and HNS sausages). Among the antibiotics, Streptomycin, Tetracycline, Gentamycin and Kanamycin resistances were the most detected: 27% of the strains were resistant to Streptomycin, 16% to Tetracycline, 14% to Gentamycin and 10% to Kanamycin. A lower number of strains were found to be resistant to other antimicrobials, in particular: 3% to Clindamycin, 3% to Chloramphenicol, 1.5% to Erythromycin and 1.5% to Ampicillin. Fermented sausages from Italy (IM1, IM2 and IAL), Slovenian sausages SN and SWO and the Spanish CB sausages showed to be colonized by susceptible lactobacilli, with no resistances to the antibiotics tested in the study.

### 3.3. Biogenic Amines (BAs) Production

Aminobiogenic potential results demonstrated high variability among the strains based on their species and source of isolation. No BA producers were detected among the *L. sakei* strains, while a high number of *L. curvatus* (26 out of 40 strains) accumulated these compounds. As already evidenced for antibiotic resistance, also the decarboxylase activity was strongly linked to the geographic origin of the isolates. The highest number of BA producing strains have been isolated from Spanish products, indicating an effect of raw materials, environmental conditions, and processes in exerting a selective pressure on microbial communities and their metabolisms (Table 4).

Among the 40 *L. curvatus* strains, 26 were decarboxylase-positive and namely 19 produced tyramine, 10 putrescine and 1 histamine, while 4 strains were able to accumulate both tyramine and putrescine (Table 4). Seven out of 26 aminobiogenic strains presented one or more antibiotic resistances, showing different traits that are related to their safety features. Apart from enterococci, *L. curvatus* is considered the main tyramine producer among LAB in fermented sausages [20,50], while *L. sakei* is usually described as non-aminobiogenic. The decarboxylase potential has been demonstrated to be strain dependent [51]. Moreover, Ladero et al. (2015) described the capability of *L. curvatus* strains to produce both tyramine and putrescine. The latter is mainly accumulated in LAB though agmatine deiminase (AgDI) pathway, rather than ornithine decarboxylase (ODC), common in Gram negative bacteria [52].

The spontaneously fermented sausages used as source of isolation of *L. curvatus* strains presented a BA concentration ranging from about 100 mg/kg to more than 1000 mg/kg, including tyramine, putrescine and cadaverine. Interestingly, in the samples where *L. curvatus* CE-27 has been found, histamine was present at a concentration of 170 mg/kg [28].

## 4. Conclusions

Fermented sausages are produced all over Europe, with a wide diversity of manufacturing techniques and organoleptic properties between different countries and even between different regions within the same country. The 15 naturally fermented MC sausages with peculiar characteristics in terms of manufacturing and ripening conditions [28], demonstrated to be a good source of interesting autochthonous *Lactobacillaceae* to be studied for their potential technological applications in the food industry. At species level, the identified biotypes did not show a consistent biodiversity, with only *L. sakei*, dominating over the rest of the species (59.6% of isolates), *L. curvatus* present in lower proportion (26.5%) and few isolates identified as *C. alimentarius* (13.9%). Anyway, results obtained are in accordance with those described in previous studies on similar types of fermented sausages [53,54,55,56]. A more consistent biodiversity could be described in terms of strain ecology, with the Spanish sausages being the richest for the number of biotypes, while Italian and Slovenian samples showed only a low percentage of strains, belonging the majority to *L*. *sakei* species.

The evaluation of the safety profile of these Mediterranean products resulted in a high incidence of *L. sakei* (50%) and *L. curvatus* (45%) resistant to antibiotics; in addition, the safety assessment allowed to define a geographical clustering of resistant biotypes: strains isolated from Italian and Slovenian natural fermented sausages showed no incidence of antibiotic resistance and a negligible production of BA; on the contrary, the highest number of antibiotic-resistant isolates were detected in Spanish and Croatian salami, with a high prevalence of MDR (Multi Drug Resistant) bacteria; in particular, Streptomycin, Tetracycline, Gentamycin and Kanamycin resistances were the most observed. We found one *L. sakei* and one *L. curvatus*, which carried five resistances to antibiotics, respectively in the ECE and HZK sausages. This aspect arises a global concern linked to the safety of ready-to-eat fermented meat products; the previous large use of antibiotics in the pig production chain has led to a change in the pig microbiome and consequently in the diffusion of antibiotic resistant genes (ARG) in the meat environment [57]. The application of good manufacturing practices in the pork industry can help to control antibiotic resistant pathogen or spoilage species, but when are technological species, such as LAB, to harbor resistant genes, this can represent a difficult risk to be monitored for the consumer safety.

Finally, amino biogenic potential seemed to be species-related; in fact, no BA producers were detected among the *L. sakei* analysed strains, while a high number of producer strains was found among *L. curvatus*. After the safety assessment, a total of 45 *Lactobacillaceae* strains (44 *L. sakei* and 1 *L. curvatus* respectively) were classified to be safe, having no resistances and amino biogenic capacity. *L. sakei* demonstrated to be the most abundant species present in naturally fermented MC sausages but also the species with the best safety traits. These results could be the starting point for improved knowledge regarding the study of the technological attributes and bioprotective activity of these strains. The main aim will be to select natural starters with added value to be employed in the fresh and fermented meat productions.

## Figures and Tables

**Table 1:** Primers for selected antibiotic resistance genes used in this study.

Primers Name	Oligonucleotide Sequence (5′-3′)	Expected Band (bp)	Positive Control Strain	Reference
**ermA1**	TCTAAAAAGCATGTAAAAGAA	645	*E. faecium* PE1	[38]
**ermA2**	CTTCGATAGTTTATTAATATTAGT			
**ermB1**	GAAAAGGTACTCAACCAAATA	639/694	*E. faecium* PE1	[38]
**ermB2**	AGTAACGGTACTTAAATTGTTTAC			
**ermC1**	ATCTTTGAAATCGGCTCAGG	275/294	*L. reuteri* 70	[39]
**ermC2**	CAAACCCGTATTCCACGATT			
**tetL1**	GTMGTTGCGCGCTATATTCC	696	*E. faecium* LMG 20927	[29]
**tetL2**	GTGAAMGRWAGCCCACCTAA			
**tetM1**	GAACTCGAACAAGAGGAAAGC	740	*L. plantarum* 146	[35]
**tetM2**	ATGGAAGCCCAGAAAGGAT			
**tetS1**	GGAGTACAGTCACAAACTCG	335	*L. reuteri* 541	[36]
**tetS2**	GGATATAAGGAGCAACTTTG			
**tetW1**	GAGAGCCTGCTATATGCCAGC	168	*L. reuteri* 534	[34]
**tetW2**	GGGCGTATCCACAATGTTAAC			

**Table 2:** Type of sausages, number of isolates and biotypes from the four different production countries (Italy, Slovenia, Spain and Croatia).

Production Country	Type of Sausage	Sample Name	Number of Isolates	Biotypes
Italy	Salame Fabriano-producer 1 (Marche)	IM1	58	3
	Salame Fabriano-producer 2 (Marche)	IM2	48	2
	Salame Alfianello (Brescia), Lombardy	IAL	67	4
Slovenia	Traditional smoked salami with nitrates	SN	70	5
	Traditional smoked salami without nitrates	SWO	70	6
Spain	Salchichòn Bérchules	ESB	69	11
	Chorizo Bérchules	ECB	48	16
	Chorizo Olvera	ECO	52	7
	Salchichon Olvera	ESO	70	19
	Chorizo Ecija	ECE	69	22
	Salchichon Ecija	ESE	69	8
	Salchichon Alhendin	ESA	67	11
Croatia	Salami ZminjskaKlobasica	HZK	53	21
	Traditional smoked salami	HS	49	5
	Traditional unsmoked salami	HNS	55	11
Total	15		914	151

**Table 3:** Unimodal distribution of MICs for *L. sakei* and *L.* *curvatus* isolated biotypes. The resistant strains for each antibiotic are highlighted (bold and italics).

Antibiotic ^a^	Species	MIC Value (µg mL^−1^)														
		<0.016	0.032	0.063	0.125	0.25	0.5	1	2	4	8	16	32	64	128	256
Gen	*L. sakei*						1	1	4	11	33	27	**10**	**3**		
	*L. curvatus*						1	3	2	9	10	10	**5**			
Kan	*L. sakei*								1	5	12	20	20	21	**5**	**6**
	*L. curvatus*								1	3	8	11	7	7	**3**	
Str	*L. sakei*								1	5	10	10	17	22	**22**	**3**
	*L. curvatus*							1		2	3	7	7	9	**4**	**7**
Tet	*L. sakei*				7	5	9	26	17	5	6	**1**		**14**		
	*L. curvatus*					2	4	3	9	9	6	**6**	**1**			
Ery	*L. sakei*	4	11	37	17	13	3	5								
	*L. curvatus*		3		7	15	7	6	**2**							
Clin	*L. sakei*		66	6	1	9	2	2	**2**	**1**	**1**					
	*L. curvatus*		20	3	11	1		2	**2**	**1**						
Chlor	*L. sakei*				7	2	10	33	29	7	**2**					
	*L. curvatus*						1	4	4	27	**3**	**1**				
Amp	*L. sakei*		7			2	9	20	12	40						
	*L. curvatus*					1	15	11	7	4	**2**					

^a^ Gen = Gentamycin; Kan = Kanamycin; Str = Streptomycin; Tet = Tetracycline; Ery = Erythromycin; Clin = Clindamycin; Chlor = Chloramphenicol; Amp = Ampicillin.

**Table 4:** BAs production by *L. curvatus* strains sorted by origin. *: the presence of (S) or (R) indicates an antibiotic-sensitive or antibiotic-resistant strain, respectively.

Countries of Origin	*L. Curvatus* Aminobiogenic Strains	Tyramine	Histamine	Putrescine	Cadaverine
Italy	*L. curvatus* IAL6 (S) *	+	−	−	−
Spain	*L. curvatus* ESB8 (S)	−	−	+	−
	*L. curvatus* ECB11 (S)	+	−	−	−
	*L. curvatus* ECB12 (S)	+	−	−	−
	*L. curvatus* ECO46 (S)	+	−	−	−
	*L. curvatus* ESO6 (R)	−	−	−	−
	*L. curvatus* ESO13 (R)	+	−	−	−
	*L. curvatus* ESO14 (R)	+	−	+	−
	*L. curvatus* ESO16 (R)	+	−	−	−
	*L. curvatus* ESO19 (R)	−	−	−	−
	*L. curvatus* ESO25 (R)	−	−	−	−
	*L. curvatus* ESO52 (R)	−	−	−	−
	*L. curvatus* ESO59 (S)	+	−	+	−
	*L. curvatus* ESO61 (R)	−	−	+	−
	*L. curvatus* ECE16 (R)	+	−	+	−
	*L. curvatus* ECE25 (R)	−	−	−	−
	*L. curvatus* ECE27 (R)	−	+	−	−
	*L. curvatus* ECE32 (S)	+	−	−	−
	*L. curvatus* ECE35 (S)	+	−	−	−
	*L. curvatus* ECE37 (S)	+	−	−	−
	*L. curvatus* ECE40 (R)	−	−	−	−
	*L. curvatus* ECE42 (S)	+	−	−	−
	*L. curvatus* ECE46 (S)	+	−	+	−
	*L. curvatus* ECE51 (S)	−	−	+	−
	*L. curvatus* ECE52 (S)	+	−	−	−
	*L. curvatus* ECE53 (R)	−	−	−	−
	*L. curvatus* ECE54 (S)	+	−	−	−
	*L. curvatus* ECE57 (S)	−	−	+	−
	*L. curvatus* ESE3 (S)	−	−	+	−
	*L. curvatus* ESE19 (S)	−	−	+	−
	*L. curvatus* ESA38 (S)	+	−	−	−
	*L. curvatus* ESA53 (S)	+	−	−	−
Croatia	*L. curvatus* HZK3 (R)	+	−	−	−
	*L. curvatus* HZK32 (R)	−	−	−	−
	*L. curvatus* HZK43 (R)	−	−	−	−
	*L. curvatus* HZK49 (R)	−	−	−	−
	*L. curvatus* HNS1 (R)	−	−	−	−
	*L. curvatus* HNS18 (R)	−	−	−	−
	*L. curvatus* HNS20 (R)	−	−	−	−
	*L. curvatus* HNS55 (S)	−	−	−	−
Total strains	40	19	1	10	0

## Data Availability

Not applicable.

## Supplementary Materials

The following supporting information can be downloaded at: https://www.mdpi.com/article/10.3390/foods11182776/s1, Table S1. List of strains used in this study with respective origin, MIC, ATB PCR detection and biogenic amines prediction.
